# To the Editors of the Medical and Physical Journal

**Published:** 1807-10

**Authors:** W. Warnock

**Affiliations:** Assistant Surgeon H. M. Ship Saturn


### *To the Editors of the Medical and Physical Journal.*

Gentlemen,

JAMES BLAKE, a seaman, aged 28, when up at the mast-head, assisting in furling the royals, fell; in consequence of which he fractured his scapula, tibia, and fibula. On being carried into the sick bay, the fractures being reduced, he was bled, and had an opiate; his pulse all this time was very full, nor did it undergo the least change by bleeding. The accident happened about six o'clock: I saw him afterwards at nine; at that time his pulse was rather more regular; he was perfectly in his senses, had not much fever, and appearances promised a speedy cure. About eleven o'clock, when one of the attendants went to see if he wanted any thing, he was found dead. On examination after death, nothing was discovered that could in the least degree have caused his death, except an extravasation about the fifth vertebra; his death must have been occasioned, in my opinion, by a compression of the medulla spinalis. No paralysis of the lower extremities was observed; on the contrary, when reducing the fracture, he complained of pain: and on my examining the fractured limb, at nine o'clock, I felt a considerable degree of warmth.

I am, &c.

W. WARNOCK,

*March* 21, 1807. Assistant Surgeon H. M. Ship Saturn.

---

